# Observations on the Injurious Effects Which Frequently Arise in Children from Protracted Suckling

**Published:** 1827-08

**Authors:** Edward Morton

**Affiliations:** Physician to the Western Dispensary, and to the Royal Metropolitan Infirmary for Sick Children.


### DISEASES OF CHILDREN.

*Observations on the injurious Effects which frequently arise in Children from protracted Suckling.* By EDWARD MORTON, M.B. L.M. Physician to the Western Dispensary, and to the Royal Metropolitan Infirmary for Sick Children.

SEVERAL cases which I had previously witnessed led me to believe, some years ago, that inflammation of the brain, or its membranes, might be produced in children in consequence of their being suckled for an undue length of time. Since that period I have had opportunities of observing infantile diseases on a much more extended scale, and, my attention having been expressly directed to the point in question, I have not only become fully convinced of the correctness of the opinion I then entertained, but have been induced to extend it still further.

The idea I conceive to be new; but this is of little consequence, if it only have truth for its basis. If this be allowed, (and I submit it is a question which experience alone can decide,) it is perhaps not improbable that it may hereafter prove extensively beneficial; since precautionary measures may be founded upon it, which, if uniformly adopted, may be the means of saving many children from falling victims to one of the most painful and fatal diseases to which they are subject.

As, however, I am anxious that the present communication should take up as small a space as possible in the valuable pages of this Journal, I will at once observe, that, from the experience which I have already had upon the subject, I am inclined to draw the following conclusions:

1st. That, if children are suckled for an undue* length of time, they will be liable to be affected, in consequence, with inflammation of the brain.

* "Undue,"—i. e. any period beyond nine or ten months.

CASES.

Case I.—Emma Lane, æt. two years, suckled for one year eleven month, admitted for cephalitis.* The mother suckled a former child for a very long period, and it died of "water on the brain."

Case II.—Edmund Power, æt. two years, still at the breast, admitted for hydrocephalus chronicus. The head is very large; fontanelles open; superficial veins large and prominent.

Case III.—Sophia Hamley, æt. one year two months, still at the breast, admitted for cephalitis.

Case IV.—William How, æt. one year six months, suckled for thirteen months, admitted for cephalitis.

Case V.—David Hepburn, æt. two years and six months, suckled for two years four months, admitted for cephalitis.

Case VI.—Samuel Hanks, æt. one year nine months, suckled for one year eight months, admitted for cephalitis.

Case VII.—Amelia Hill, æt. two years six months, suckled for one year nine months, admitted for cephalitis.

2dly. That the same effect will take place in infants if suckled by women who have been delivered an undue† length of time, although the infants themselves may not have been at the breast for too long a period.

CASE.

Case VIII.—Ellen Willoughby, æt. nine months, admitted for cephalitis; suckled by a woman who has been delivered one year nine months.

3dly. That, if they should not become affected with the disorder in question during or soon after the time they are thus improperly suckled, they will nevertheless acquire thereby a predisposition to cephalic disease at some future period of their lives.

CASES.

Case IX.—Sarah Strickling, æt. four years, suckled for one year six months, admitted for cephalitis. The mother of this child lost three previously with "water in the head:" all these were suckled for more than eighteen months.

Case X.—George Speering, æt. four years, suckled for one year six months, admitted for cephalitis,

Case XI.—Ann Archer, æt. seven years, suckled for three years, admitted for hydrocephalus: died.

* Cephalitis."—As this term will be made use of in the relation of the following cases, I beg leave to say that I mean by it those symptoms which give rise to, and precede, the disease strictly and properly called Hydrocephalus, or water in the brain, and which are well described by Dr. Yeats.

† See the above conditional sense in which I employ this term.

Case XII.—Cornelius Leary, æt. six years, suckled for one year six months, admitted for cephalitis: died.

Case XIII.—Sophia Peverel, æt. three years, suckled for two years, admitted for cephalitis.

Case XIV.—Maria Turley, æt. four years, suckled for one year three months, admitted for cephalitis: died. This child had a previous attack about six months before, from which she recovered while under my care.

Case XV.—Robert Selkirk, æt. three years six months, suckled for one year one month, admitted for cephalitis. The mother lost two children previously, one with "inflammation of the brain," and the other of convulsions: both of these were suckled for more than one year.

Case XVI.—Eliza Ferreira, æt. five years, suckled for one year seven months, admitted for cephalitis. The mother lost another child previously of "water in the head," at the age of one year five months, which was suckled until it died.

And 4thly and lastly, that children who are suckled for an undue length of time, when labouring under other diseases, will be much more liable to have the head secondarily affected than other children.

CASES.

Case XVII.—Sarah Swann, æt. four years six months, suckled for one year, admitted for hooping-cough with convulsions.

Case XVIII.—Mary Harris, æt. two years three months, suckled for one year four months, admitted for hooping-cough with an affection of the head.

Case XIX.—Maria Hughes, æt. two years, suckled for one year three months, admitted for convulsions after hooping-cough. The mother lost another child previously with "water in the head," which was suckled for fourteen months.

Case XX.—Thomas Benson, æt. one year six months, suckled for one year four months, admitted for pneumonia, with an affection of the head.

Case XXI.—Mary Kenner, æt. six years, suckled for one year six months, admitted for hooping-cough, with well-marked affection of the head.

Case XXII.—John Ennes, æt. one year seven months, suckled for one year, was admitted for bronchitis; afterwards a decided affection of the head supervened.

With respect to the manner in which protracted lactation produces the complaint above mentioned, I am at present undecided, but think it most probable that the affection of the head is consequent upon derangement in the chylopoietic viscera, which may possibly arise from the milk becoming so far deteriorated in quality as to be unfit any longer for the purposes of nutrition. This opinion seems to be strengthened by Case VIII. in which it appeared that it was not any particular state of the child which produced the cephalic affection, but the impoverished condition of the nurse's milk.

In conclusion I beg to observe, that it was my intention to have waited until I might have been able to collect a greater mass of evidence than I at present possess upon the subject in question, before I published my opinions thereon; but as individual experience, however extensive, can never be entitled to general confidence, I have, upon further consideration, deemed it more advisable at once to submit the foregoing observations and cases to the profession, in order that the conclusions which I have felt myself warranted in drawing from them may be either corrected or confirmed by the experience of others.

I think it right to add, that, upon referring to the total number of cases of cephalic inflammation that have come under my care since I have been in the habit of making particular inquiries on the subject, I find that a *very large majority* have occurred in children who had been suckled for an improper length of time.

15, *Eaton-street, Grosvenor-place;*

*June* 8*th*, 1827.

